# Mapping the Patient’s Journey in Healthcare through Process Mining

**DOI:** 10.3390/ijerph17186586

**Published:** 2020-09-10

**Authors:** Michael Arias, Eric Rojas, Santiago Aguirre, Felipe Cornejo, Jorge Munoz-Gama, Marcos Sepúlveda, Daniel Capurro

**Affiliations:** 1 Department of Business Computer Science, Universidad de Costa Rica, San Ramón 111-4250, Costa Rica; 2 Department of Clinical Laboratories, School of Medicine, Pontificia Universidad Católica de Chile, Santiago 8320000, Chile; eric.rojas@uc.cl; 3 Department of Industrial Engineering, School of Engineering, Pontificia Universidad Javeriana, Bogotá 110231, Colombia; saguirre@javeriana.edu.co; 4 Department of Computer Science, School of Engineering, Pontificia Universidad Católica de Chile, Santiago 7820436, Chile; facornejo@uc.cl (F.C.); jmun@uc.cl (J.M.-G.); marcos@ing.puc.cl (M.S.); 5 School of Computing and Information Systems, University of Melbourne, Victoria 3010, Australia; dcapurro@unimelb.edu.au

**Keywords:** process mining, healthcare, customer journey maps

## Abstract

Nowadays, assessing and improving customer experience has become a priority, and has emerged as a key differentiator for business and organizations worldwide. A customer journey (CJ) is a strategic tool, a map of the steps customers follow when engaging with a company or organization to obtain a product or service. The increase of the need to obtain knowledge about customers’ perceptions and feelings when interacting with participants, touchpoints, and channels through different stages of the customer life cycle. This study aims to describe the application of process mining techniques in healthcare as a tool to asses customer journeys. The appropriateness of the approach presented is illustrated through a case study of a key healthcare process. Results depict how a healthcare process can be mapped through the CJ components, and its analysis can serve to understand and improve the patient’s experience.

## 1. Introduction

Customer journey mapping (CJM) [1] is a well-known technique that focuses on assessing how customers interact with a company or organization. This allows the gain of a deeper knowledge about the customers’ perceptions, and becomes an essential tool to improve the overall customer experience (CX). This knowledge can be used to generate competitive advantage and satisfied customers [2]. Understanding each step required to complete a process (customer paths), and how customers interact with a company’s services throughout that journey are key aspects to assess customer needs, the ability to improve them, and the delivery of great products and services. During a journey, customers can interact with a company through different methods or channels (e.g., on-line, phone call, etc.), go through different stages, and face specific business context factors. Recently, companies in several industries embarked in a transformation to take advantage of the benefits of new digital technologies. This digital transformation may impact key business factors, customer relationships, as well as products, services and processes [3]. However, a primary limitation is that companies do not have enough knowledge about their customers or how their end-to-end processes look like [4].

Along with this digital transformation, and the data generated through digital interactions, two novel disciplines have emerged: process science and data science [5]. Process science combines knowledge from information technologies and management sciences, to run and improve operational processes (e.g., time or cost aspects) based on a model-driven perspective. Within this discipline, Business Process Management (BPM) [6]—a process-oriented discipline—can be used to enhance organizational processes, aiming to combine different approaches to execute the design, implementation, control, monitoring, and optimization of business processes. Data science seeks to turn data into real value (data-driven approach), by focusing on generating knowledge to improve processes from larger and smaller datasets [7].

As part of the process science discipline, there is an important data-oriented sub-discipline called process mining (PM), which can be used to enhance companies’ operational processes. The aim of PM is to extract knowledge about process execution from data stored in existing information systems [8]. This sub-discipline can be understood as a bridge between process science and data science. Process mining has been used in several industries including healthcare [9] and education [10], as well as in resource allocation or recommendations for business processes [11].

In healthcare, there is a particular interest in a correct and efficient execution of both clinical and business processes. However, it is widely known that healthcare processes are often challenging due to their complexity, dynamics, and sometimes owing to their ad-hoc nature and multiplicity of disciplines involved [12]. Hospitals usually embrace standards to ensure the quality of healthcare processes. As a consequence, the identification of typical and non-typical patient journeys, and the monitoring compliance (process deviations) with hospital policies and standards become a focus of interest. Disciplines such as process mining could be used to support those initiatives and provide valuable insights not only to improve our understanding of clinical processes, but also to influence their execution and efficiency [9].

In this paper, we use customer journey analysis as a method to map patients’ experience using process mining, which in turn guides the creation of key indicators to improve the patients’ satisfaction, based on multiple identified touchpoints during a specific healthcare process. We conduct a case study in which the CJM has been put into practice in two different scenarios based on an Emergency Room process.

## 2. Background

### 2.1. Process Mining in Healthcare

The provision of quality care to patients is the most relevant aspect in a healthcare process [5]. Process can either have a positive or negative impact on patient outcomes. Process mining provides the capability to study healthcare processes using data routinely stored in the electronic health records (EHRs). This can provide details about executed activities, their sequence, duration, and resources involved [9]. Using this information, stakeholders in healthcare environments can analyze and improve clinical processes. The order in which tasks can be performed and its resource allocation can be modified to improve clinical outcomes; clinical processes can be checked against recommended clinical guidelines to ensure adherence to best practices.

Process mining has been applied in several healthcare domains, using different techniques and diverse datasets, and on multiple clinical settings [9]. Process mining has shown good results in healthcare and its application can potentially be expanded to include additional uses.

### 2.2. Customer Journey Mapping

Improving the customer’s experience (CX) has become one of the priorities in numerous business contexts and industries [13]. According to a study by McColl-Kennedy et al. [2], there are multiple definitions of CX, but the authors highlight the importance of focusing on elements that create value (e.g., resources, activities, context, interactions, customer role), cognitive responses, and discrete emotions at different touchpoints throughout the customer’s interactions.

**Figure 1:**
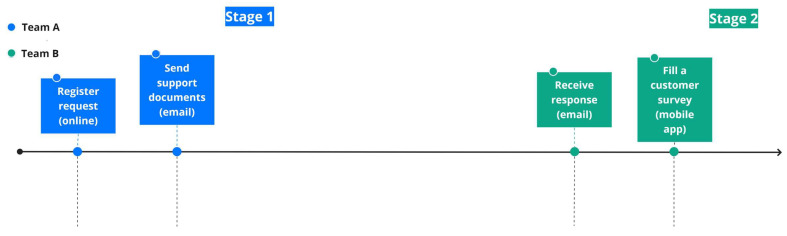
Example of the sequence of frequent activities in a customer service process.

Customer Journey Mapping (CJM) has gained popularity among researchers and practitioners as a tool to obtain knowledge about customers and to understand better the customer experience. A CJM is a device that allows researchers to attain a deeper comprehension about experiences, the generation of specific metrics, and the understanding of how they interact throughout the different stages required to provide a product or service [14]. A CJM is a visual representation that shows the sequence of events that emerge during the interaction between a customer and a specific process. For example, in a customer service process, the customer interacts with several touchpoints and through different channels such as creating an on-line request, emailing support documents, receiving an email response, and completing a customer satisfaction survey using a mobile app (see Figure 1). Throughout the whole process, the customer engages with each activity (touchpoints), which can help the people involved in the process to understand all different stages in a journey that a customer may undertake.

Primarily, CJMs allow organizations to track customer interactions and to inform decision-making processes on how they could be enriched [15].

### 2.3. Components of CJM (Customer Journey Mapping)

In a recent study [14], the authors conducted a literature review to clarify the most common components of CJM. The main components identified are: customer, journey, mapping, goal, touchpoint, timeline, channel, stage, experience, lens, and multimedia. Those components and their corresponding description are explained in Section 3.

These components can be used to create a blueprint for a company’s products or services, giving companies a more comprehensive view about their work methods, processes, required information, technology used in their operations, and factors that could emerge in specific domains. These components can serve as an important technique to elicit the evaluation of the experience throughout the journey. They can help to identify less important touchpoints, to reveal what customer actually do, instead of what process’ owners think they do, to assess customer experience, and to promote strategic initiatives, among other uses [1].

CJM has been applied in multiple domains to improve existing products and services or to create new ones. Uses include mobile services [16], marketing [15], and libraries [17].

There are other process-oriented methods available not only to investigate customers’ interactions but also to assess their experiences based on customer interviews (CIT—Critical Incident Technique) [18], surveys considering the sequence of the customer touchpoints (SIT—Sequential Incident Technique) [19], or performing service walkthroughs (STA -Service Transaction Analysis-) [20]. Nevertheless, all of them involve the collection of new data. CJM using process mining can become a data-driven alternative to efficiently map customer journeys.

## 3. Process Mining in Healthcare as a Customer Journey Mapping Tool

According with Terragni and Hassani [21], only few studies have applied process mining in costumer journey analysis, making it an interdisciplinary field with great opportunity to conduct research in this background. Particularly, they proposed a novel approach for applying process mining techniques to web log customer journey analysis. Bernard and Andritsos [14] were the first authors to propose the application of process mining for a CJM, highlighting the potential of this discipline to analyze customer journeys. The authors proposed a customer journey exploration map using event logs and data analytics [22] as an opportunity to characterize hundreds or thousands of customer journeys at the same time. Bernard and Andritsos extended their work in [23], and developed a javascript-based tool that can handle process mining models to create a CJM at different levels of granularity; and additionally, they introduced the use of genetic algorithms to solve the problem of automatically building CJMs from event logs [24].

Process mining and patient journey has been studied before [25,26], but further studies are needed to fully understand the experience of the patient. Therefore, in this paper we address this research gap. In Section 2.3, each of the main components of CJM (based on [14]) has been mentioned. In this section, those components are now mapped to each of their corresponding concepts in the healthcare domain and what they can mean for a process mining perspective as follow.

**Customer**: as we stated, the customer is the stakeholder who undertakes the journey. Considering the variety of existing healthcare processes and the people involved in their execution, three different customers categories can be identified [9,27]: patients, healthcare professionals, and healthcare administrators.

**Journey:** a customer journey should contain at least one journey. An event log [5] can be defined as a set of traces, each encompassing all the events performed in a particular process instance or a case. Journey Maps can be discovered from information stored in event logs. Moreover, a journey can be part of any healthcare process such as inpatient episodes [28]), outpatient visits, diagnostic test paths, telemedicine paths, and online patient portal paths [27].

**Mapping:** Process mining techniques are used in multiple industries, including healthcare, to create process maps [29]. There are dozens of discovering algorithms that focus on the control-flow perspective supported by distinct process mining tools; such as, ProM [30] (e.g., Heuristics Miner, and Fuzzy Miner), Disco [31] (interactive process map) and Celonis [32] (the flow of most frequent activities known as happy path).

**Goal:** allows, both organization and customer, to establish a common target to analyze with more detail the customer journey. It obtains the process ’As-Is’ representation, recognizes time constraints, identifies process bottlenecks, performs an organizational analysis (resource perspective), detects process deviations, reduces process complexity, among others, are typical examples of outcomes that can be obtained when applying process mining techniques [5] to analyze specific customer journey goals. From the healthcare point of view, the clinical outcome is the most relevant goal. These outcomes have a direct impact in changing the patient’s long-term quality of life.

**Touchpoint:** is a set of interactions (events) where a patient might be involved during the healthcare process (journey). Those interactions may include events such as: diagnose, treat, follow-up, and the application of preventative measures, thus with the intention of improving clinical outcomes [9].

**Timeline:** refers to an end-to-end timestamp [5] registered in order to track when the distinct touchpoints occur and it’s order.

**Channel:** depicts the method chosen by the customer to participate with the different touchpoints identified previously. Typical examples are: (i) Hospital Information Systems/Process Information Systems [33]/Electronic Patient Records (EPR) [34]/Electronic medical records (EMR); (ii) Patient Portal (Medical history, schedule appointments, message their physician, view bills and make payments all online) (iii) ePrescribing (sending prescriptions to pharmacies); among others.

**Stage:** touchpoints could be grouped into a cluster of activities (defined as stages or phases). Distinct techniques could be used to split the process into “sub-processes”, much like, using the own semantics of the process (decomposition manually), using Passages, applying Single-Entry Single-Exit (SESE) decomposition, or using generic decomposition approaches [35]. For instance, in a healthcare process, a cluster of “Registration” events or “Treatment Decision” events, as well as “Inpatient” and “outpatient” events could be identified and grouped.

**Experience:** aims to capture and evaluate stakeholder’s experience (e.g., healthcare professionals and patients) of different services. Positive or negative perceptions can be obtained through several mechanism such as: experts feedback [36], patients feedback using some of the multiple experience surveys available [37], quality of life and other patient centered measures (e.g., patient satisfaction, patient perceptions, patient engagement, patient participation, and patient preferences), staff observations, shadowing, video recording [38]. Using process mining techniques would allow process owners to monitor and analyze identified journeys, and to improve the user experience (e.g., identify Key Performance Indicators (KPIs) and metrics to increase patient satisfaction).

**Lens:** is a domain’s specific component that can be added and would represent particular elements of the process landscape. Lens might differ across different process scenarios. Some common examples are: environmental factors taking into account during healthcare process execution (e.g., pollution, weather, exposure to ultraviolet radiation), food hygiene, pests and vectors (e.g., mosquitoes), unhealthy housing, and waste management.

**Multimedia:** similar to other industries, important data can be extracted from different multimedia content that registered how the distinct process touchpoints are executed. Video recording [38], audio, multicast, and broadcast, are examples of traditional media that can be represented, stored, and transformed into event logs.

## 4. Case Study

To understand the importance of the application of CJM using process mining, a case study with two distinct scenarios have been carried out for different diagnostics. The first case study is for patients with Pneumonia, and the second case study is for patients with Acute Myocardial Infarction.

### 4.1. Context and Description

Both cases were conducted using historical data collected in a 500-bed teaching hospital located in Santiago, Chile and the study was approved by the institutional ethics committee (Id: 180504002). The historical data was extracted from the Hospital Information System and the Emergency Department’s Electronic Medical Record. Multiple activities can be executed on both settings (in the clinic or the Emergency Room), therefore the prefix “ER” will be used to differentiate them. Furthermore, it is relevant to mention that the project was reviewed and authorized by the hospital’s local human subject review board.

### 4.2. Data and Event Log Construction

The data was extracted in CSV format, and encompassed information about clinical episodes that took place between 2 January 2017 and 30 June 2018. The data includes 6715 cases, including 523,521 events executed, and eight main process activities. We found detailed information about patient demographics, clinical episodes, diagnoses, and timestamped process activities.

To create the event log, we considered the following features:

- Episode ID: corresponds to the episode identifier.
- Activity: refers to the phases that involved patient’s interactions with the clinical services.
- Episode diagnosis: patient diagnosis documented by the physician.
- Activity details: further details about the episode diagnosis registered by the physician.
- Timestamp:date of the activity performed.
- Age: patient’s age at the moment of the episode.
- Gender: patient’s gender.

**Figure 2:**
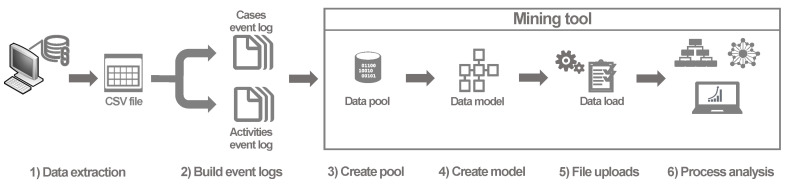
Steps followed for data processing and process analysis.

To load the information into the selected process mining tool (see further details in Section 4.3), we executed a set of previous steps (see Figure 2) to perform a data configuration procedure to generate a proper data model which in turn allows to perform a detailed process analysis.

After the data was extracted we split it into two CSV files. The first file is identified as the event log that contained the case identifier plus some attributes (“Cases_EventLog”). In this event log the following fields were included: EpisodeID, gender, age, and episode diagnosis. Table 1 shows an extract of the “Cases_EventLog”.

The second event log ("Activities_EventLog") contains information about the process activities. We included following fields: EpisodeID, activity, timestamp and activity details. Table 2 shows an extract of the “Activities_EventLog”.

Third, we uploaded both files into a data pool and, fourth, we created a new data model. This step involved adding the two files mentioned above, establishing the activity table and selecting the following columns: CaseId column (EpisodeID), activity column, and timestamp column. To finish the data model creation step, a entity-relation diagram was created. The EpisodeId was selected as a Foreign key between the source table (“Cases_EventLog”) and the target table (“Activities_EventLog”). Once the data model was created, the data was loaded into the analysis tool. Finally, in our sixth step, we execute the process analysis for both case studies presented in Section 4.4 and Section 4.5.

### 4.3. Process Mining Tool

There are several tools that offer distinct techniques and algorithms to perform process mining analysis. For the case studies of this article, the Celonis platform [32] was selected. Celonis is a leader in Enterprise Performance Acceleration process mining software. This commercial platform has recently been renewed into an Intelligent Business Cloud and now provides a set of features grouped by distinct categories like Business Process Analytics, Action Engine, Event Log Collection, Machine Learning and Artificial Intelligence, and Process Automation, among others. We have chosen this tool mainly due to its capacity to handle complex processes together with the feature of being able to generate the most common path that cases follow—also known as “happy-path”—which not only helps us to identify touchpoints between the patient and the process, but it also visualizes and explores all the variants that have been discovered in the process. In addition, we have decided to take advantage of the process analytics features and capabilities that Celonis offers, in order to generate a set of analytic visualizations which helped us to explore the process in a more interactive way and to create valuable process KPIs regarding the selected episodes as explained below.

### 4.4. Case Study I: Pneumonia

In this first case study, process mining was used to find the patients’ journey map when they are diagnosed with Pneumonia.

#### Process Models and Journey Analysis

**Figure 3:**
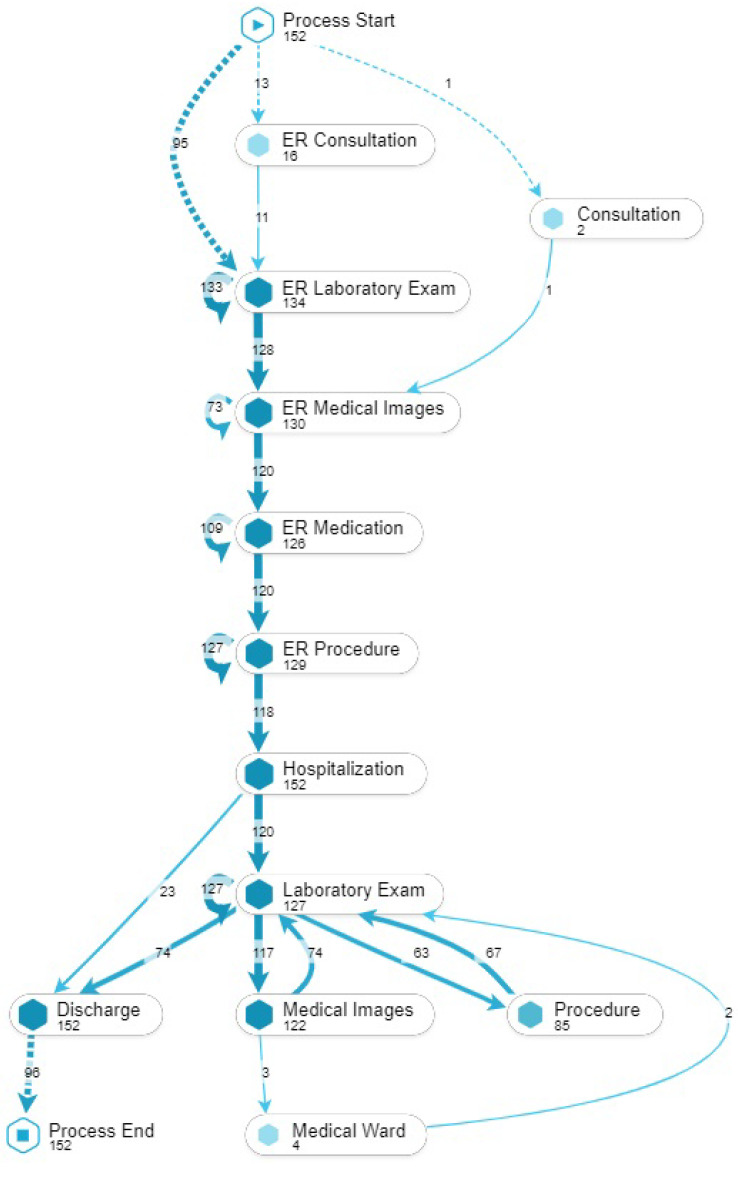
Pneumonia patients’ journey map.

**Figure 4:**
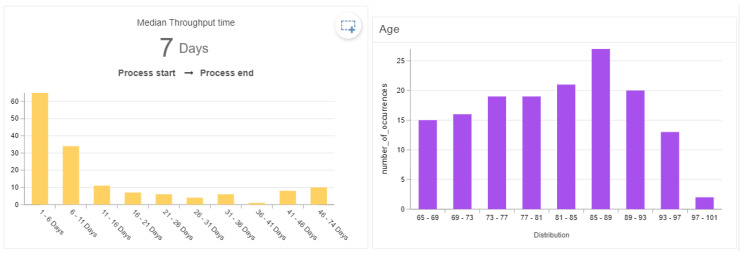
Length of stay of patients who were 65 years of age or older.

A process discovery algorithm was used to obtain the model that shows the different touchpoints and the patients’ journey that is presented in Figure 3. In total there are 12 touchpoints where most of the cases (97 percent) start in the Emergency Room (ER) where laboratory exams, medical images and procedures are performed. Given the nature of the dataset, all patients required hospitalization and presented a median length of stay of 7 days (see Figure 4).

**Figure 5:**
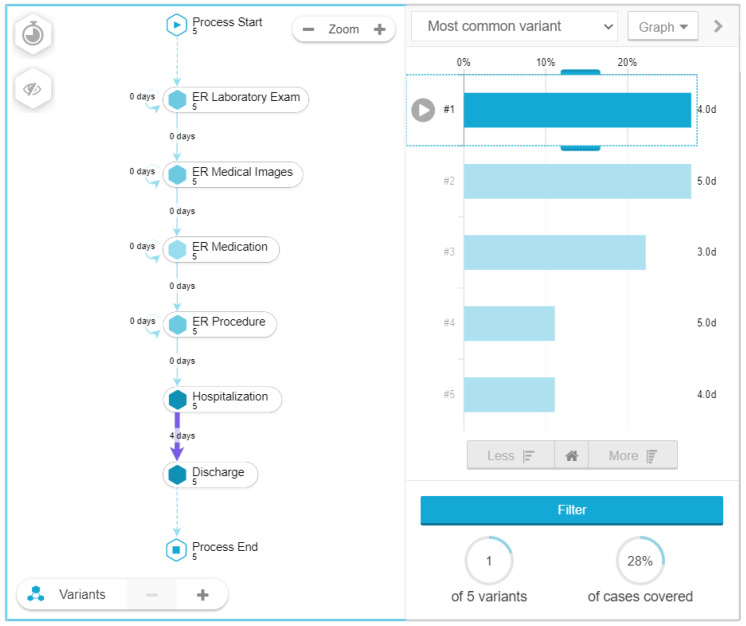
Variant analysis for pneumonia patients.

A variant analysis was used to discover the different paths that patients go through on the healthcare process and their relationship with the length of stay. In the case of pneumonia the most common variant covers 28 percent of the total cases and has six touchpoints as shown in Figure 5: ER (laboratory, images, medication, procedure) hospitalization and discharge with a medium length of hospitalization stay of 4 days.

**Figure 6:**
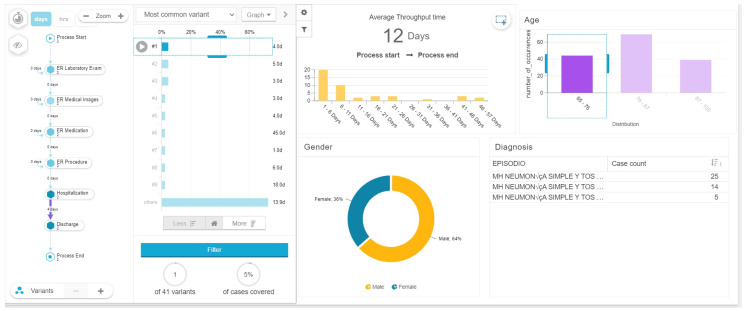
Length of stay of patients between 65 and 76 years of age.

**Figure 7:**
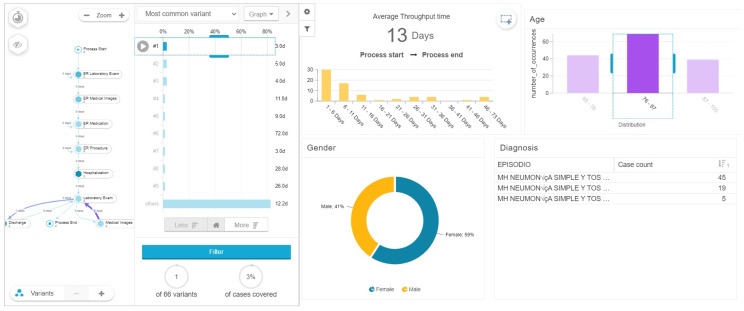
Length of stay of patients between 76 and 87 years of age.

**Figure 8:**
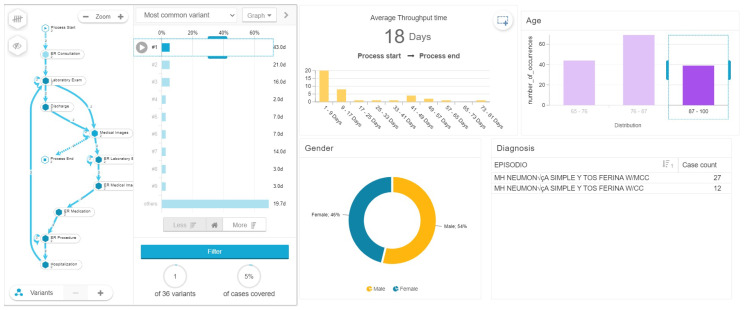
Length of stay of patients between 87 and 100 years of age.

With this analysis we found a relationship between the age, gender, length of stay and complexity of the process model, measured by number of variants. Patients between 65 and 76 years old, most of them men (64 percent) have an average of 12 days of hospital stay with 41 process variants (see Figure 6) in comparison, patients between 76 and 87 years old with a majority of woman (59 percent) had an average length of stay of 13 days with 66 process variants (see Figure 7), while patients between 87 and 100 years old with a majority of men (54 percent), had the highest average length of stay with 18 days among the three groups analyzed, and 36 process variants (see Figure 8). This highlights that older patients require additional touchpoints like medical images, laboratory exams and procedures, which can be translated into different patient journeys. Additionally, we found that the prevalence rate is first higher in the men’s age group of 65–76 with a 64%; then it switches over to the women’s age group of 76–87 with a 59%, and then higher again among the men’s group, this time those between 87 and 100 years old (see Table 3).

### 4.5. Case Study II: Acute Myocardial Infarction (AMI)

In this second case study, process mining was used to find the patients’ journey map when they are diagnosed with an AMI.

#### Process Models and Journey Analysis

**Figure 9:**
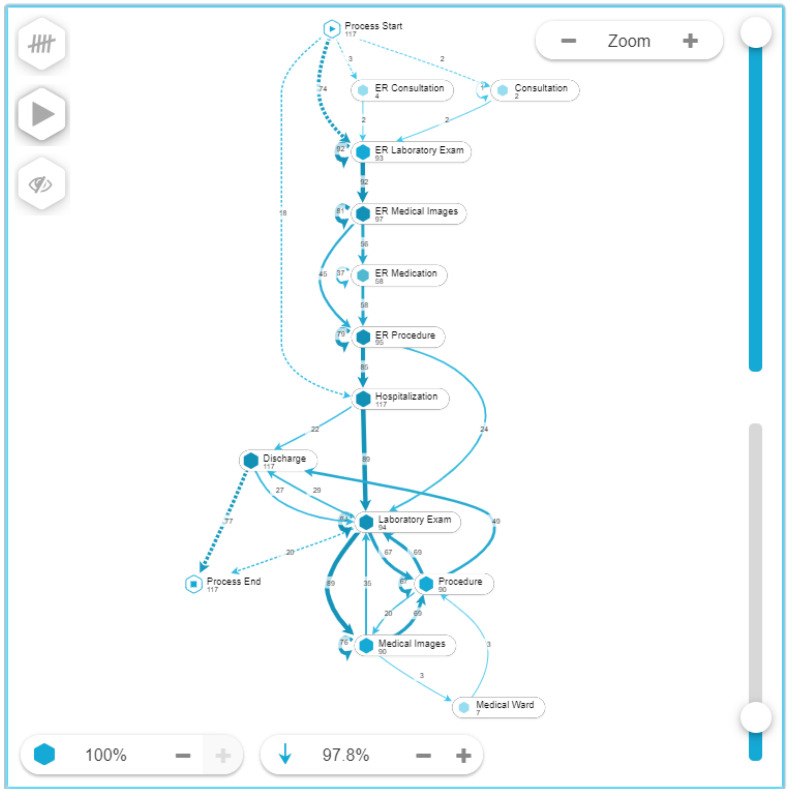
AMI patients’ journey map.

In this case the patient’s journey also starts in most of the cases (65 percent) with ER. In comparison with the pneumonia’s journey where most of the exams and medical images are performed in the Emergency Room, in the case of hearth stroke the exams and procedures are performed before or after the procedures that follows hospitalization as Figure 9 shows.

**Figure 10:**
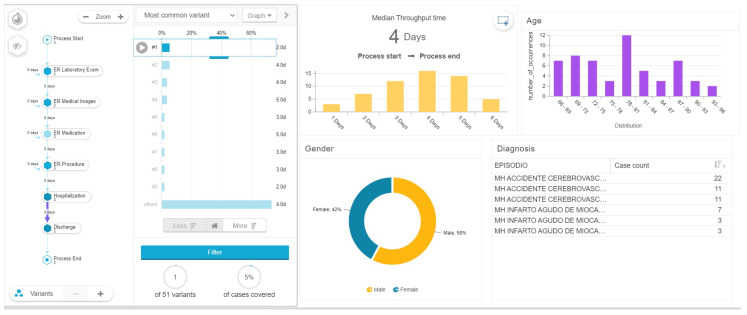
Variant and short stay for AMI patients.

A relationship was found between the hospital length of stay and the number of variants and touchpoints. In cases with length of stay between 1 and 7 days, the number of variants is 50 and the most common path has five touchpoints as seen in Figure 10.

**Figure 11:**
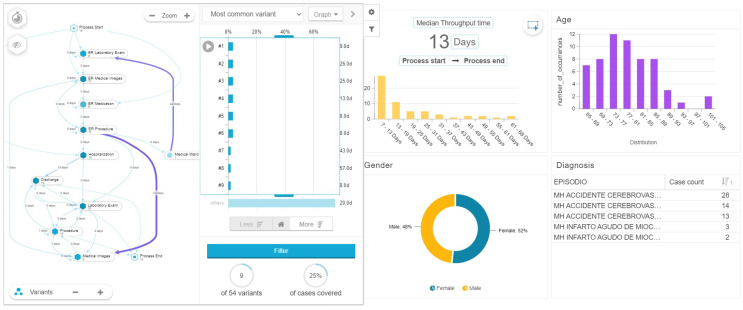
Variant and extended stay for AMI patients.

Cases with lengths of stay longer than 7 days tend to have more variants, in this case 54 and the path that covers 25 percent of observed cases has nine touchpoints it the patients journey (Figure 11).

### 4.6. Lead to Key Indicators

A patient’s experience during healthcare is a key aspect of healthcare quality. Developing a visual display that presents how the process is executed and characterizing it according to distinct attributes would help to monitor the process more effectively and enable the implementation of improvement measures. Healthcare organizations should constantly seek to assess the patients’ perception of the services they receive. This dimension was not included in the customer journeys assessed since patient satisfaction was not available in the studied dataset. However, the inclusion of standard patient satisfaction surveys in CJM activities is strongly encouraged. As an example, the HCAHPS (Hospital Consumer Assessment of Healthcare Providers & Systems) survey assesses several dimensions of the patient’s experience [39]:

- Communication with healthcare professionals;
- Responsiveness of hospital staff;
- Operation of hospital units;
- Cleanliness of hospital facilities;
- Quiet environment;
- Discharge information;
- Amenities provided.

The assessment of those indicators (among others) would help clinicians and administrators to identify where the changes are required, to proactively test them and to assess the impact of those changes, and to determine the most effective ways to improve patient care, operational efficiency, and enhance process outcomes.

## 5. Discussion

CJM has become an interesting and relevant management technique with a clear set of concepts to understand and describe process paths from the customer point of view. One of the most relevant aspects is the capacity to provide a context for the different variables that affect the customer experience.

The use of process mining to generate customer journey maps has created a powerful analysis tool to understand how processes variants (paths) and touchpoints may affect the customer experience, as well to discover how end-to-end processes look like.

Specifically, in healthcare processes the concepts of CJM can be directly applied, contextualizing them in a much better and detailed way, so as to provide complementary information for the analysis of these processes. Adding complementary information will also allow users to include patients’ feedback to improve and enrich clinical processes. In the same way, it also allow healthcare experts to describe processes, not only using a conceptual process model, but also using real process paths and variants (what customers really do), including more frequent and less frequent touchpoints, analyzing process performance, and identifying process deviations. Based on a more detailed description, it becomes easier to discover process improvement opportunities.

When combining the application of CJM technique and process mining discipline on a real-life case study of a clinical process in a hospital in Santiago, Chile, it has become clear that routinely collected clinical data has the capability to reveal—with great detail— the multiple interactions (touchpoints) that a patient has when visiting the hospital. The CJ maps can be quickly generated from electronic healthcare record data and become a valuable visual tool that allows a detailed analysis on how patients interact during the attention process (end-to-end process), according to specific diagnoses, which may provide important details about the analyzed healthcare processes.

In addition to discovering the process using real-world-data, these methods can help to establish gaps between the expected and the real patient experience. Comparing the two case studies, it was found that the trajectories vary depending on the diagnosis, which serves as a useful input to understand relevant touchpoints and where the process could be improved.

The approach proposed in this article is novel as it is aimed at understanding the interaction between the patient and the emergency department at each touchpoint, using a systematic approach based on CJM. In [25], the control-flow perspective is analyzed, i.e., in which order patient care is performed in an emergency room. In [28], it is analyzed how different healthcare professionals interact to care for patients. In this article, these views are complemented, also taking into account the patient’s perspective. In [26], the customer journey is analyzed in a simplified and ad-hoc way; instead, the approach proposed in this article uses a systematic approach based on CJM techniques.

A key aspect in analyzing healthcare processes and generating key indicators is how the discovered model can be complemented with additional information. In both of our case studies, we were able to add demographic patient data such as age and gender. Adding more information to the study will broaden the possibilities of generating tailored and valuable indicators for each patient type.

One of the challenges that arises when using process mining to understand the CJM is to find the most effective method to measure the patient’s experience in order to link it with specific process paths. Typically, organizations obtain customer feedback by means of surveys, based on samples that lacks of the necessary respond rate to be reliable.

## 6. Limitations

In our study, we identified some limitations. First, the dataset did not include the outpatient component of the patient’s journey. Having access to the whole care continuum should provide a richer picture of the complete patient journey. Second, patient satisfaction surveys were not available in the included dataset, which limited our ability to assess the association between specific process variants and patient satisfaction. This dimension will be addressed in future work. Finally, in the presented case studies, the Lens and Multimedia components have not been evaluated since the event logs were generated from electronic medical records which only capture the clinical dimensions of the process.

## 7. Conclusions and Future Work

This article describes how to apply process mining techniques to understand patient journeys in healthcare, providing a new and complementary perspective for understanding and improving patient care. The proposed approach allows to analyze touchpoints in the lights of the patient and to understand the multiple variants or paths that appear in the customer journeys and how they could be related to the patient’s experience. It can be used to deal with complex processes, including those with multiple trajectories. We have been able to conduct a case study where we have applied the CJM technique to a real-life healthcare process, encouraging the creation of healthcare KPIs as an alternative to assess how healthcare units are achieving their goals in order to provide improvements on quality of care.

As future work, we would like to explore the application of additional process mining algorithms and techniques in order to gain knowledge from the control-flow perspective, but also from other process perspectives, such as the organizational perspective, to analyze the experience of a patient at each touchpoint and the interactions with clinical staff. Further, expanded case studies should be carried out, including the complete care continuum, to see the proper use of this vision and adjust the concepts if necessary. The incorporation of patient satisfaction represents a key challenge to work on.

## Figures and Tables

**Table 1:** Example of an event log at case identifier level.

EpisodeID	Gender	Age	Diagnosis
24	Female	70	Simple pneumonia and whooping cough
388	Female	71	Pleural effusion and pneumonia
480	Male	80	Acute Myocardial Infarction

**Table 2:** Example of the an event log at activity level.

EpisodeID	Activity	Timestamp	Activity Details
24	ER Laboratory Test	30/01/2017	Fast aerobic blood culture
	ER Laboratory Test	30/01/2017	Rapid determination of anti-HIV antibodies
	ER Procedure	30/01/2017	Blood culture collection
388	Laboratory Test	21/02/2018	Hemoglobin
	Laboratory Test	21/02/2018	Thromboplastine
	Laboratory Test	21/02/2018	Blood chemistry
480	ER Procedure	07/05/2017	Blood vein extraction
	ER Procedure	07/05/2017	Phleboclysis installation

**Table 3:** A comparative summary of different pneumonia groups analyzed.

Age Group	Average Length of Stay	# of Variants	Gender %
65–76	12	41	Female 36%; Male 64%
76–87	13	66	Female 59%; Male 41%
87–100	18	36	Female 46%; Male 54%

## Abbreviations

The following abbreviations are used in this manuscript:

AMI	Acute Myocardial Infarction
BPM	Business Process Management
CIT	Critical Incident Technique
CJ	Customer journey
CJM	Customer Journey Mapping
CORFO	Chilean National Development Agency
CX	Customer experience
EHR	Electronic health Record
EMR	Electronic medical records
EPR	Electronic Patient Records
ER	Emergency Room
HCAHPS	Hospital Consumer Assessment of Healthcare Providers & Systems
KPI	Key Performance Indicators
PM	Process mining
SESE	Single-Entry Single-Exit
SIT	Sequential Incident Technique
STA	Service Transaction Analysis
